# Owner-reported treatments and outcomes of perceived injuries to the thoracic and pelvic limb of agility dogs

**DOI:** 10.3389/fvets.2024.1409199

**Published:** 2024-08-22

**Authors:** Bianca M. Alva, Arielle Pechette Markley, Abigail Shoben, Nina R. Kieves

**Affiliations:** ^1^VCA Animal Referral Center of Arizona, Mesa, AZ, United States; ^2^Department of Veterinary Clinical Sciences, College of Veterinary Medicine, The Ohio State University, Columbus, OH, United States; ^3^College of Public Heath, Division of Biostatistics, The Ohio State University, Columbus, OH, United States

**Keywords:** agility, dog, injury, thoracic limb, pelvic limb, treatment, outcome

## Abstract

**Objective:**

The aim of this study was to identify the type of veterinary care sought by handlers of injured agility dogs, the types of treatments the dogs received, and the timeframe for return to training and competition.

**Procedures:**

Owners of agility dogs completed an internet-based survey. They were instructed to report injuries that had kept the dog from training or competing for over a week, identify which area(s) of the body had been injured and answer questions about the most severe injury to each body part. Additional questions included if handlers had sought veterinary care, who primarily determined treatment, type of treatment(s), and length of time before the dog could return to full training and competition.

**Results:**

This sample included data on 1,714 total injuries from 1,256 unique dogs. Handlers sought veterinary care for over 80% of injuries across all anatomical locations. Handlers were most likely to seek specialty veterinary care for reported injuries to the stifle (71%), iliopsoas (63%) and tibia (61%), and least likely for reported injuries to the carpus (34%), metatarsus (33%) and metacarpus (22%). Treatment of reported injuries to the antebrachium and stifle were most likely to be directed by a veterinarian (>70%), while reported injuries of the thigh (51%) and hip (53%) were least likely. Rest was the most common treatment for all injuries. Return to sport within 3 months was common (>67%) for most perceived injury locations, though dogs with reported stifle injuries took longer to return to competition and had a higher rate of retirement.

**Conclusion and clinical relevance:**

Owners of agility dogs have a high rate of seeking veterinary care for injuries. Overall return to sport rates were high, with the stifle being the notable exception. Future studies regarding specific treatment of injuries in agility dogs, and how injuries and their treatment affect return to agility after injury are required to provide optimal care protocols for these canine athletes.

## 1 Introduction

Canine agility is one of the most popular dog sports worldwide and participation has grown, with entries into sponsored events by the American Kennel Club increasing 38% from 2009 to 2019 (1). There has also been an increase in reported injuries in agility dogs, with an overall injury rate of 41.7% reported in 2019 compared to 32% reported the previous decade (2, 3). The increased injury rate, as well as the increasing participation in the sport, require refined knowledge of injury treatment and outcomes to provide the best care recommendations for these patients.

Numerous retrospective survey studies have reported on types of injuries reported by owners of agility dogs. These studies have shown that reported shoulder injuries are common, with back injuries also commonly reported (2–6). Reported overall return to competition timelines have varied from relatively quick resolution with 71% returning to agility in less than 4 weeks in one study (4) and 50% returning in less than 1 month in another (3), to relatively longer resolution with only 26% returning in less than 3 weeks and 33% taking longer than 8 weeks to return to competition (6). However, none of these studies have examined return to agility timelines by type of injury.

A handful of these studies have also provided limited information about treatment of injuries among agility dogs. The reported percentage of dogs being treated by any veterinarian varied from 41 to 78% (3, 4, 6). One previous study reported the frequency of various therapies among all injured agility dogs, but this information was not specific to the type of injury (6). Given the growth of sports medicine and rehabilitation as a veterinary specialty, as well as increased availability of additional treatment modalities such as orthobiologics, this study sought to characterize the types of professionals consulted, as well as the types of treatments used by agility handlers when their dog has a perceived injury keeping it from agility training and competition. Given the variety in return to sport timelines in previous studies, we also aimed to provide further description of return to sport timelines among agility dogs specific to the perceived anatomic location of the injury. Having a baseline of timeframe for return to agility following specific injuries may help guide the practitioner in developing expectations for clients as to how long the period of convalescence may be when their dog is injured.

Given the paucity of information specific to perceived injury location in the literature, the objectives of this study were to describe the type of veterinary care sought by handlers of injured agility dogs, the types of treatments the dog received, and the timeframe for return to agility training and competition.

## 2 Materials and methods

### 2.1 Study design

Data were acquired from an internet-based survey that was distributed primarily via social media during a 6-week period in 2019 (2). Individuals were eligible if they had at least one dog competing in agility in the past 3 years. Dogs were not required to have an injury for handlers to complete the survey. If handlers had more than one dog that was eligible, alphabetical order using the name of the dog was used to select the dog for which the survey was completed. The research protocol and survey were reviewed and approved by The Ohio State University Institutional Review Board.

Information about the survey has been previously published in detail (2). Briefly, handlers were asked if their dog ever had an injury that kept them from training or competing in agility for greater than 1 week. If the answer was yes, they were asked to identify all locations on the body where they believed the dog had been injured, based on a diagram illustrating and naming anatomic regions (2). Specific questions regarding each injured anatomical region were then asked. If the dog had experienced more than one injury to the same anatomical region, owners reported information for the injury that had kept the dog out of agility training and competition for the longest period of time.

Questions specific to the injury included what type(s) of care had been sought, who primarily determined treatment, the type of treatments utilized, and the length of time before the dog could return to full agility training and competition. The following options were provided regarding type of veterinary care sought: primary care, veterinary specialist, chiropractor, and other; owners were instructed to check all that applied. Similarly, owners were asked to check all that applied for options regarding treatment pursued: rest, medication, home rehabilitation, formal rehabilitation, regenerative medicine, surgery, and other. If surgery was selected, a follow up question asked owners to select the type of surgery from a pre-populated list specific to each anatomical region or write in the type of surgery if not listed. Owners were also asked who primarily determined treatment: a veterinarian, another professional (chiropractor, massage therapist, etc.), an agility trainer or friend, or the owner themself.

### 2.2 Statistical analysis

The percentage of owners endorsing each option for type of veterinary care sought (if any) and each treatment option was calculated for each anatomical region and 95% confidence intervals for these proportions were calculated using the Wilson score interval. As a descriptive study, no formal statistical testing was done to compare these percentages across regions. To characterize the reported length of time to return to competitive agility by anatomical region, dogs that were reported to be still undergoing treatment were excluded (as the time of the original injury was not available). Then the percentage of dogs who returned to sport within 1 month, within 3 months, within 6 months, within 1 year, and after more than a year were calculated, along with the percentage of dogs who were retired. This paper details injuries reported to be sustained to the thoracic limb (metacarpus, carpus, antebrachium, elbow and shoulder), as well as those to the pelvic limb (hip, iliopsoas, thigh, stifle, tibia, tarsus, and metatarsus). All statistical analyses were conducted using Stata v15.1.

## 3 Results

The sample included data from 4,197 dogs. This paper reports data on 1,714 total injuries from 1,256 unique dogs (some dogs contributed data on more than one injury).

**Figure 1:**
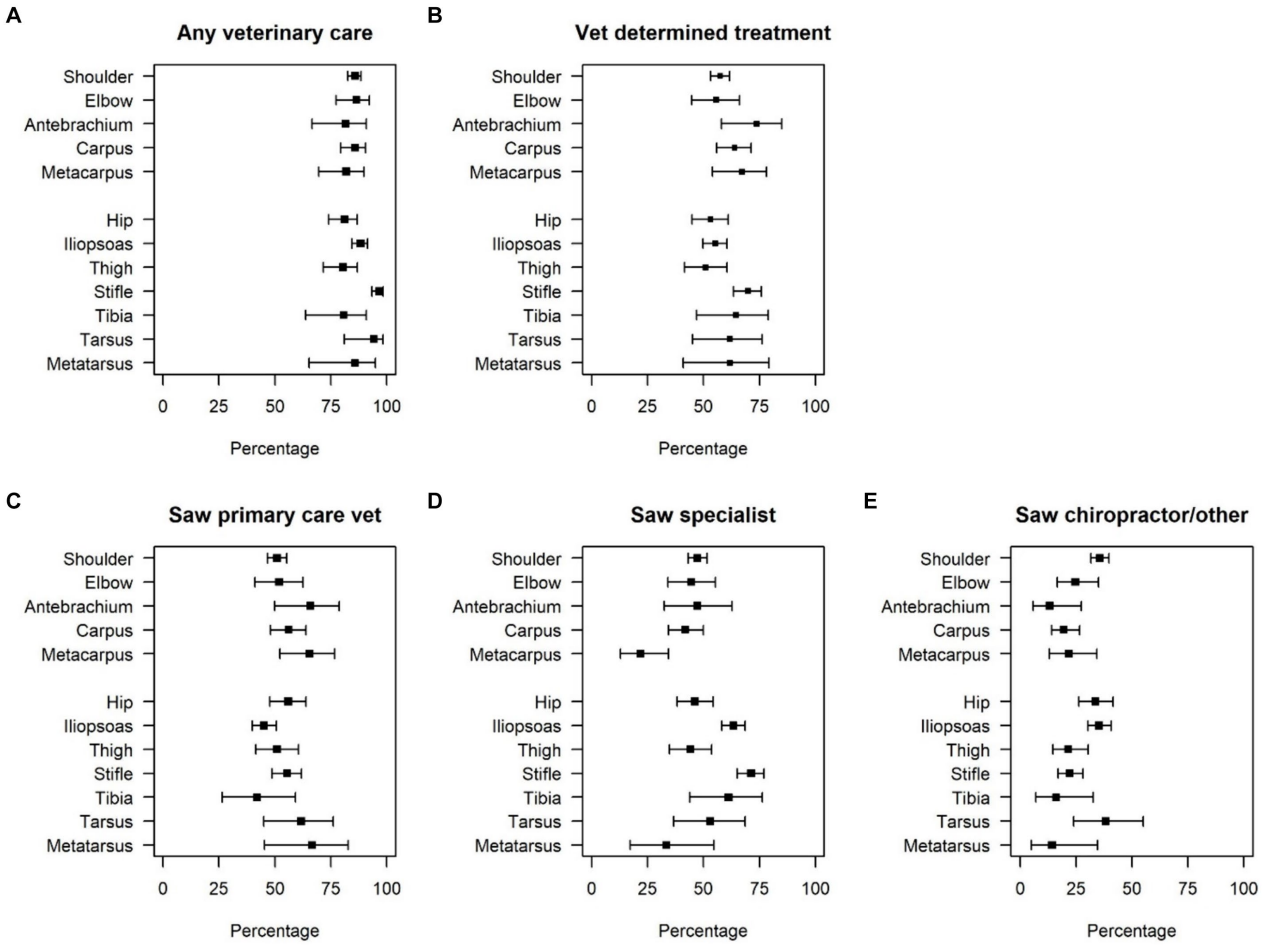
Type of veterinary care sought by owner-reported injury location. Bars indicate 95% confidence intervals for the estimated proportions. Panel **(A)** shows the percentage of dogs that sought any veterinary care, panel **(B)** shows the percentage of dogs in which a veterinarian determined treatment, panel **(C)** shows the percentage of dogs that sought primary veterinary care, panel **(D)** is the percentage of dogs that saw a veterinary specialist and panel **(E)** shows the percentage of dogs that sought care from a chiropractor/other.

Across all anatomical regions, owners reported seeking veterinary care for a large majority of injuries (range 80–97%, Table 1; Figure 1A). Owners were most likely to seek any veterinary care (97%) and specialty veterinary care (71%) for reported stifle injuries. Owners were also likely to seek specialty veterinary care for reported iliopsoas (63%) and tibia (61%) injuries (Table 1; Figure 1B). Owners stated that a veterinarian primarily determined treatment for over 50% of injuries across all reported locations (range: 51–74%, Table 1; Figure 1C), with percentages exceeding 70% for the stifle and antebrachium (Figures 1D,E).

**Table 1:** Type of veterinary care sought by owner-reported injury location.

Injury location	*N* ^a^	Veterinary care was sought	Saw primary care veterinarian	Saw veterinary specialist	Saw chiropractor / other	Veterinarian primarily determined treatment
*Thoracic Limb*						
Shoulder	522	448 (86%)	266 (51%)	247 (47%)	186 (36%)	300 (57%)
Elbow	81	70 (86%)	42 (52%)	36 (44%)	20 (25%)	44/79 (56%)
Antebrachium	38	31 (82%)	25 (66%)	18 (47%)	5 (13%)	28 (74%)
Carpus	148	127 (86%)	83 (46%)	62 (34%)	29 (16%)	94/147 (64%)
Metacarpus	55	45 (82%)	36 (65%)	12 (22%)	12 (22%)	37 (67%)
*Pelvic Limb*						
Hip	143	116 (81%)	80 (56%)	66 (46%)	48 (34%)	76 (53%)
Iliopsoas	326	288 (88%)	147 (45%)	207 (63%)	115 (35%)	180 (55%)
Thigh	102	82 (80%)	52 (51%)	45 (44%)	22 (22%)	52 (51%)
Stifle	213	206 (97%)	118 (55%)	152 (71%)	47 (22%)	149 (70%)
Tibia	31	25 (81%)	13 (42%)	19 (61%)	5 (16%)	20 (65%)
Tarsus	34	32 (94%)	21 (62%)	18 (53%)	13 (38%)	21 (62%)
Metatarsus	21	18 (86%)	14 (67%)	7 (33%)	3 (14%)	13 (62%)

a With available treatment data.

**Figure 2:**
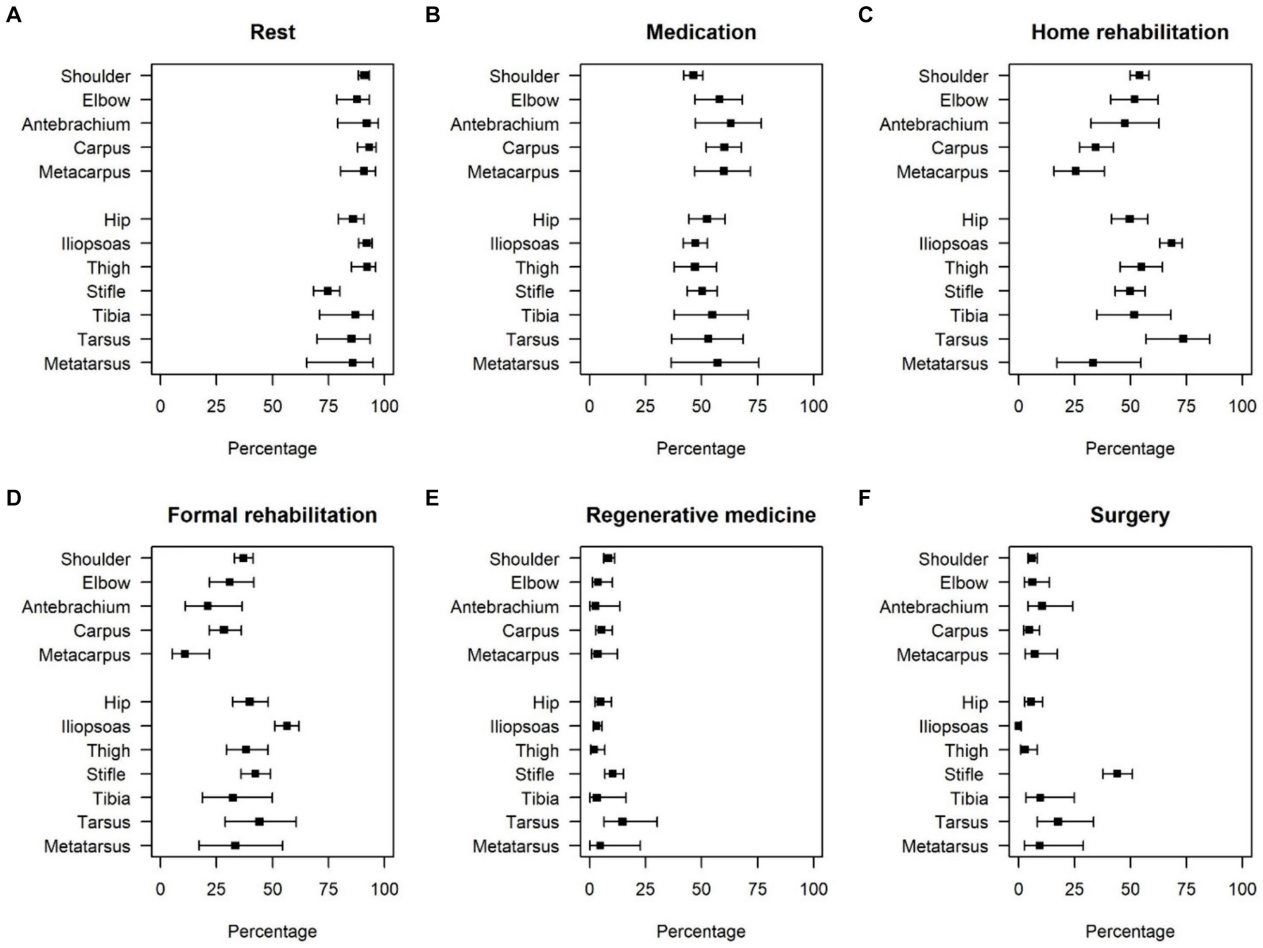
Types of treatment pursued by owner-reported injury location. Bars indicate 95% confidence intervals for the estimated proportions. Panel **(A)** shows the percentage of dogs that underwent rest, panel **(B)** shows the percentage of dogs where medication was chosen as treatment, panel **(C)** shows the percentage of dogs that underwent home rehabilitation as treatment, panel **(D)** shows the percentage of dogs that underwent formal rehabilitation as treatment, panel **(E)** shows the percentage of dogs that underwent treatment in the form of regenerative medicine, and panel **(F)** shows the percentage of dogs that underwent surgery as treatment.

Reported treatments were generally similar across all injuries to the pelvic and thoracic limbs (Table 2; Figure 2). Rest was the most common treatment reported (above 85% for all except stifle, Table 2; Figure 2A), and medication use was also similar across all locations (46–60%, Table 2; Figure 2B). There was greater variation in the percentage of owners reporting both home and formal rehabilitation as a treatment based on perceived injury location. Both formal and home rehabilitation were most frequently utilized for reported iliopsoas and tarsal injuries (Table 2; Figures 2C,D). Regenerative medicine and surgery were infrequent treatments for all locations, except surgery was common (44%) for reported stifle injuries (Table 2; Figures 2E,F). Of the 94 dogs who had surgery for treatment of their reported stifle injury, owners stated that 63 (67%) had a corrective osteotomy for cranial cruciate ligament (CCL) rupture. The remainder were stated to be luxating patella correction (*n* = 15; 16%), lateral suture stabilization for CCL rupture (*n* = 9; 10%), or another surgery (*n* = 7; 7%).

**Table 2:** Types of treatment pursued by owner-reported injury location.

Injury location	Rest	Medication	Home rehabilitation	Formal rehabilitation	Regenerative medicine	Surgery
*Thoracic Limb*						
Shoulder	475 (91%)	242 (46%)	282 (54%)	193 (37%)	44 (8%)	31 (6%)
Elbow	71 (88%)	47 (58%)	42 (52%)	25 (31%)	3 (4%)	5 (6%)
Antebrachium	35 (92%)	24 (63%)	18 (47%)	8 (21%)	1 (3%)	4 (11%)
Carpus	138 (93%)	89 (60%)	51 (34%)	42 (28%)	8 (5%)	7 (5%)
Metacarpus	50 (91%)	33 (60%)	14 (25%)	6 (11%)	2 (4%)	4 (7%)
*Pelvic Limb*						
Hip	123 (86%)	75 (52%)	71 (50%)	57 (40%)	7 (5%)	8 (6%)
Iliopsoas	300 (92%)	154 (47%)	223 (68%)	184 (56%)	10 (3%)	0 (0%)
Thigh	94 (92%)	48 (47%)	56 (55%)	39 (38%)	2 (2%)	3 (3%)
Stifle	159 (75%)	107 (50%)	106 (50%)	90 (42%)	22 (10%)	94 (44%)
Tibia	27 (87%)	17 (55%)	16 (52%)	10 (32%)	1 (3%)	3 (10%)
Tarsus	29 (85%)	18 (53%)	25 (74%)	15 (44%)	5 (15%)	6 (18%)
Metatarsus	18 (86%)	12 (57%)	7 (33%)	7 (33%)	1 (5%)	2 (10%)

**Figure 3:**
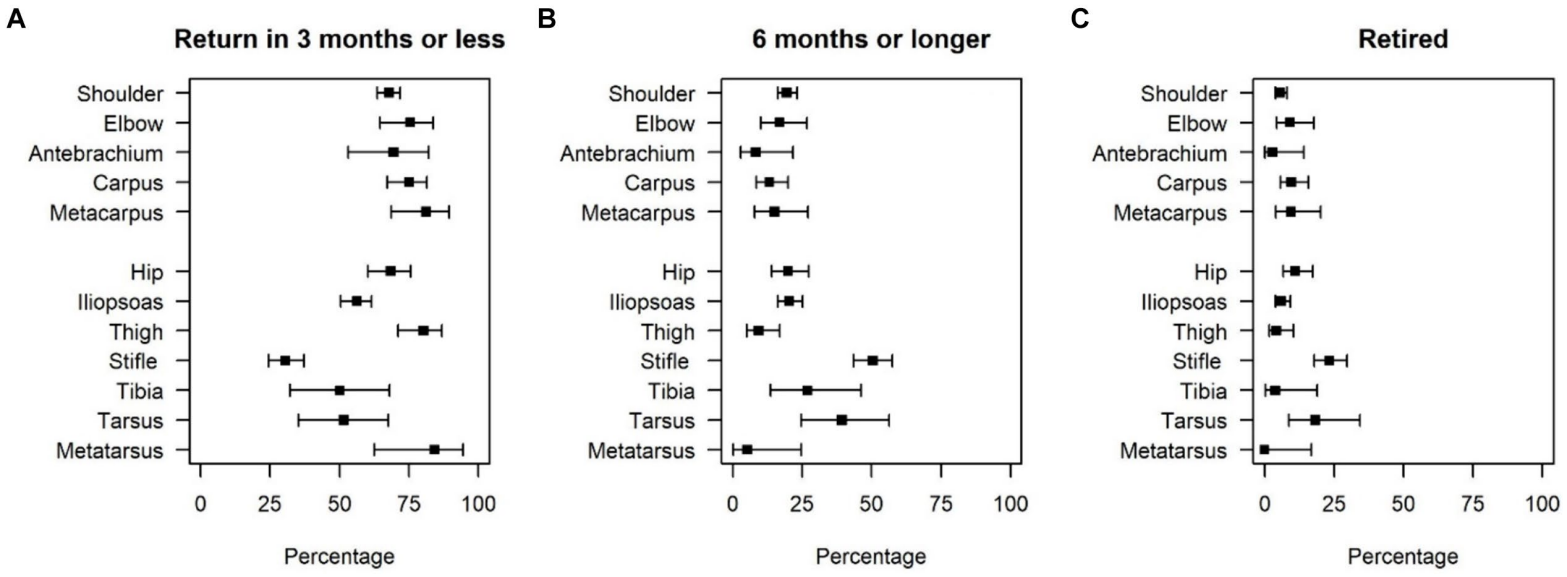
Percentage of dogs returning to training and competition by owner-reported injury location within different time frames. **(A)** Shows the percentage of dogs returning within 3 months, **(B)** shows the percentage of dogs who took more than 6 months or were retired, and **(C)** shows the percentage of dogs who were retired.

Return to training and competition within 3 months was common (>67%) for most perceived injury locations (Table 3; Figure 3A). Prolonged convalescence (>6 months or retired) was noted for injuries reported to be to the iliopsoas, stifle, tibia, and tarsus, with reported stifle injuries having the longest time to return to sport (Table 3; Figure 3B). Retirement rates were low (11% or lower) for all locations except stifle (23%) and tarsus (18%) (Table 3; Figure 3C).

**Table 3:** Time to return to competition by owner-reported injury location.

Injury location	*N* ^a^	<1 month	1–3 months	4–6 months	6–12 months	>1 year	Retired
*Thoracic Limb*							
Shoulder	485	165 (34%)	164 (34%)	62 (13%)	47 (10%)	20 (4%)	27 (6%)
Elbow	77	27 (35%)	31 (40%)	6 (8%)	4 (5%)	2 (3%)	7 (9%)
Antebrachium	36	17 (47%)	8 (22%)	8 (22%)	1 (3%)	1 (3%)	1 (3%)
Carpus	136	63 (46%)	39 (29%)	16 (12%)	3 (2%)	2 (1%)	13 (10%)
Metacarpus	53	30 (57%)	13 (25%)	2 (4%)	1 (2%)	2 (4%)	5 (9%)
*Pelvic Limb*							
Hip	136	50 (37%)	43 (32%)	16 (12%)	9 (7%)	3 (2%)	15 (11%)
Iliopsoas	301	51 (17%)	118 (39%)	71 (24%)	32 (11%)	11 (4%)	18 (6%)
Thigh	96	35 (36%)	42 (44%)	10 (10%)	5 (5%)	(0%)	4 (4%)
Stifle	194	24 (12%)	35 (18%)	37 (19%)	32 (16%)	21 (11%)	45 (23%)
Tibia	26	6 (23%)	7 (27%)	6 (23%)	4 (15%)	2 (8%)	1 (4%)
Tarsus	33	4 (12%)	13 (39%)	3 (9%)	3 (9%)	4 (12%)	6 (18%)
Metatarsus	19	11 (58%)	5 (26%)	2 (11%)	1 (5%)	0 (0%)	0 (0%)

a With treatment resolved (those who reported that treatment was ongoing are excluded).

## 4 Discussion

This study describes veterinary care, treatment and outcomes following injury to the thoracic and pelvic limbs as reported by owners of agility dogs. This population of handlers sought veterinary care for 80–97% of reported injuries in their agility dogs, regardless of perceived anatomical location. These percentages are higher than previous reports from North America and Finland where veterinary care for agility dog injuries was sought 40–80% of the time (3, 4, 6). Differences may be due to variability in the wording and definitions in questions related to injuries, selection bias in the surveys, or changes in mindset of handlers regarding treatment of injuries over time. This study asked owners to report on the most serious injury to each specific location, whereas Inkila et al. (6) studied all injuries within a calendar year, and thus likely reported on a larger percentage of minor injuries as compared to this study. Perceived minor injuries may have influenced the handlers to not seek veterinary care, resulting in the lower percentage of owners seeking veterinary evaluation overall. The increased percentage of handlers seeking veterinary care in 2019, as compared to the 2009 survey by Levy et al. (4), and 2013 survey by Cullen et al. (3) could be a result of the growth of sports medicine and rehabilitation specialty care. Canine rehabilitation became increasingly popular in Europe and the United Kingdom in the 1980’s, with North America closely following in the 1990’s with recognition by the AVMA of the American College of Veterinary Sports Medicine and Rehabilitation in 2010 as the newest specialty in veterinary medicine (7). Thus, the development of sports medicine and rehabilitation as a specialty recognized by the American and European specialty colleges has likely increased awareness of sport related injuries among agility handlers.

In this study, handlers sought specialty veterinary care most commonly for reported stifle injuries (71%). The higher rate of specialty care pursued for reported stifle injury compared to others may be due to the nature of stifle injury. The most reported stifle injury was cranial cruciate ligament (CCL) injury, for which advanced surgical intervention is often the treatment of choice, as surgical treatment results in better outcomes than conservative management, with corrective osteotomies reported to have a better outcome than other surgical procedures such as extracapsular stabilization (8–11). Given the advanced skills necessary to perform an osteotomy stabilization, many of these cases are referred to specialists for care. In contrast, reported distal limb injuries were least frequently seen by a specialist, with only 22% of metacarpal injuries and 33% of metatarsal injuries seen by a specialist. This may be due to the comfort level of primary care veterinarians in treating distal extremity injuries, as many only require rest and do not often require advanced diagnostics or treatment.

Reported iliopsoas injuries were also seen frequently by veterinary specialists (63%). Definitive diagnosis of this injury requires advanced imaging such as musculoskeletal ultrasound or magnetic resonance imaging (MRI), which are most commonly available in specialty practices. It is also often a diagnosis of exclusion of underlying pathology. Evaluation can be time consuming and challenging, and as such these cases are often referred (12–15). Iliopsoas injuries are most commonly treated with rest in combination with formal rehabilitation and are rarely treated surgically (16). Therefore, it is not unexpected that our study showed that rest was the most common treatment, followed by home rehabilitation and formal rehabilitation.

Interestingly, only 47% of dogs with reported shoulder injuries were seen by a veterinary specialist. The most reported specific shoulder injuries were biceps tendinopathy, supraspinatus tendinopathy, and medial shoulder instability, all of which also require advanced imaging, such as musculoskeletal ultrasound, MRI, or arthroscopy for definitive diagnosis (2, 17–20). It is unknown why fewer handlers of dogs with shoulder injuries seek specialty veterinary care compared to other complex soft tissue injuries like iliopsoas injuries given their complexity in obtaining a definitive diagnosis and treatment required for return to sport.

Conservative treatment options in this survey included rest, medication, home rehabilitation, and formal rehabilitation. Rest was the most reported treatment among all reported locations of injury. Medication was also a prevalent treatment for all injury locations, with reported use in 46–60% of cases. This may be due to the fact that most injuries sustained by agility dogs are soft tissue injuries that historically have been treated with rest and medications (4, 21). Among the thoracic limb injuries, home and formal rehabilitation was most often pursued for reported shoulder injuries. As noted earlier, the most commonly reported injury to the shoulder was biceps tendinopathy representing about 19% of shoulder injuries in agility dogs (2). Treatments reported to result in improvement in biceps tendinopathy include extracorporeal shockwave therapy and rehabilitation therapy exercises, so the higher rates of rehabilitation therapy in shoulder injuries is not surprising (18, 22). For pelvic limb injuries, home and formal rehabilitation were most frequently sought by handlers for reported iliopsoas injuries. While there is minimal research evaluating response of iliopsoas injuries to rehabilitation therapy, it is generally accepted that rest and rehabilitation therapy are the treatments of choice (14, 16, 23).

Regenerative medicine was most often pursued for injuries reported to be to the shoulder (8%), stifle (10%) and tarsus (15%), but overall use was low. Orthobiologics, such as platelet rich plasma (PRP), are frequently utilized in human athletic injuries, such as hamstring, anterior cruciate ligament (ACL), and ankle injuries, and have shown shorter return to play rates as compared to those athletes that did not receive platelet rich plasma as a part of their treatment plan (24–27). Based on this survey, the use of regenerative therapies is not yet a mainstay of therapy in canine agility injuries. Initial retrospective studies have reported improvement in shoulder and stifle injuries with orthobiologics, however prospective data is lacking (28–30). If prospective studies demonstrate improved outcomes and return to sport after injury in canine athletes, it is likely that regenerative medicine will become a more common treatment modality.

The ability and the time needed to return to sport is often of utmost concern to the agility handler. In this study, reported return to agility training and competition rates were high (89% or above) across all anatomic regions except stifle (77%) and tarsus (82%). The only previous report described an overall rate of return to agility after injury of 67%, with a decreased rate of return to agility competition in surgically treated dogs (61%) as compared to conservatively treated dogs (70%), among all injuries (31). The higher return to sport rates in our study may reflect increased access to specialty care and improved treatment options, but may also reflect differences between the samples due to differences in recruitment and injury definition. What was not ascertained via this survey is the number of owners who returned to sport with clearance from a veterinarian. Owners may have elected to return to sport sooner than recommended, or without veterinary oversight at all.

The high rate of retirement among reported stifle injuries was not surprising, as previous studies have noted a very high rate of retirement (35–48%) following surgical correction of CCL injury (31, 32). Among the 72 dogs in our sample who underwent any surgical correction of CCL injury, 22 (31%) did not return to training or competition, with the retirement rate among the dogs who underwent osteotomy qualitatively lower (18/63; 29%) than those who underwent lateral suture stabilization (4/9; 44%). Previous studies regarding outcomes after surgical treatment of CCL rupture in the general canine population show that patients who undergo a tibial plateau leveling osteotomy (TPLO) have an excellent return to function and secondary high owner satisfaction (8, 33, 34). However, outcome and return to agility competition-level function following surgical treatment of the stifle is much lower than the reported success rates in the general dog population. When comparing ACL tears in elite human athletes, a recent meta-analysis estimated the return to sport at a similar sports performance level is 83% (35), which is higher than we observed for canine athletes with CCL tears. This is likely due to the differences in pathophysiology of cruciate ligament disease between humans and canines. Cruciate disease in dogs is typically degenerative in nature, with a smaller percentage of cases being traumatic, whereas ACL tears in humans are predominantly traumatic in origin (36). Given the differences in pathophysiology, canine ligamentous repair has not proven successful and stabilization using osteotomy-based procedures like TPLO are the standard of care, whereas standard of care in humans is primary ligamentous repair followed by extensive physical therapy (37, 38). Due to the differences in pathophysiology and repair between human and canines, it is possible that canine athletes have more chronic and degenerative changes and secondary osteoarthritis compared to their human counterparts, thereby resulting in more challenges with returning to competition-level sport. However, this is difficult to infer as the prevalence of osteoarthritis in this patient population is not known. Furthermore, the rate of meniscal injury concurrently seen with cruciate injury in dogs is significantly higher than that of humans (39). This could also impact ability to return to high level sport post-injury. The percentage of dogs receiving post-operative rehabilitation therapy is also likely significantly lower than in humans, which could also contribute to the lower rates of returning to sport-level functionality. Although rehabilitation therapy has been noted to benefit patient outcome post TPLO by increasing muscle mass and stifle range of motion, it is not currently standard of care and the frequency of post-operative rehabilitation therapy is unknown in the canine population (36, 40–44).

The high rate of retirement following reported tarsal injury was unexpected. When evaluating this survey population, most of the reported tarsal surgical procedures were due to fracture (2). Racing greyhound athletes have a high rate of tarsal injuries and fractures that typically require retirement from the sport, with greater than 29% of dogs retiring following surgery (45). The higher rate of retirement and reduced ability to return to sport after tarsal injury is likely due to the complex nature of the tarsal joint and sequelae associated with injury such as degenerative joint disease, which is typically not well tolerated in the tarsal joint, particularly in highly competitive athletic dogs (45).

In contrast to the stifle and tarsus, handlers in this study reported a 94% return to sport among dogs after a reported shoulder injury, which is similar to previous studies (30). No prospective studies have assessed what type of treatment is ideal for shoulder injuries, with treatments ranging from formal rehabilitation therapy alone, to a variety of regenerative medicine treatments, and surgical intervention (29, 30, 46, 47). Shoulder injuries in human athletes in sports such as baseball have low rates of return to play and return to prior performance. One study compared surgical and non-surgical treatments for baseball pitches and reported similar return to play rates (39 and 40%, respectively) and slightly higher return to prior performance in those treated non-surgically (7% vs. 22%) (48). Return to prior performance rates in human baseball athletes compared to dogs is likely substantially different due to distinct differences in the functionality of the shoulder joint between the two species. The shoulder joint in dogs has an important function in weight bearing unlike the shoulder joint in humans (49). Additionally, the human shoulder joint has significantly increased mobility when compared to the canine shoulder joint, which is limited due to muscular and tendinous attachments medially limiting range of motion (49). Although further prospective studies are needed to evaluate return to agility for canine patients with shoulder injuries, there has been noted improvement in return to sport using orthobiologics and rehabilitation therapy following shoulder injuries (29, 46).

An important factor when considering rate of retirement for any injury is that handlers are responsible for deciding if and when their dog returns to agility training and competition following injury. They are also responsible for deciding whether training or competition variables, such as level, organization, and jump height are adjusted. Ultimately, they are also responsible for when the dog is returned to training and sport following injury. This study did not ask about performance prior to or after injury treatment, nor did the survey ask the specific reason for retirement. Thus, it is a limitation of this study that handlers may have decided to retire injured dogs due to variables unrelated to the physical inability to continue to participate as a sequela to the injury or treatment outcome.

Other limitations of this study include potential inaccuracies related to participant recall and the reporting of injuries that were not diagnosed or confirmed by a veterinarian. However, as one of the primary aims of this study was to characterize the percentage of times handlers were seeking veterinary care for perceived injuries, self-report is the only option. It is possible, however, that there was differential misclassification of the injured region, with handlers perceiving the locations of injuries to some anatomic locations better than others. Even among injuries seen by veterinary professionals, specific treatment information and definitive diagnoses were not available due to a lack of access to veterinary records. Given the nature of the available data, we did not assess potential associations between treatments and return to sport outcomes. Return to sport is dependent on the severity of injury, regardless of treatment, and our study had no information on the severity of the initial injury. Therefore, it is highly likely that severity would significantly confound the association between treatments received and return to sport, and thus these associations were not assessed in this study. Prospectively collected data with details on injury severity, are needed to assess the impact of treatments on return to sport outcome. Future studies should also assess reinjury rate, and how this differs based on treatment. Selection bias in a convenience sample survey is inherent and therefore this sample may not perfectly reflect the overall agility population. Despite the limitations of this study, the data reported provide valuable insight into how handlers of agility dogs seek care, how injuries in agility dogs are treated, and the variation in return to sport times among these dogs. These data can help inform prognosis for return to sport, though they should be used cautiously. The low return to sport after stifle and tarsal injury suggests that additional studies are needed regarding these injuries, aiding in improvement of treatment strategies. Additionally, this survey indicates the need for future prospective studies evaluating expected return to agility for specific injuries and how treatment approaches affect sport-specific outcomes.

## Data availability statement

The raw data supporting the conclusions of this article will be made available by the authors, without undue reservation.

## Ethics statement

Ethical review and approval was not required for the animal study because the Ohio State University Office of Responsible Research Practices determined the project was exempt from IRB review as it was an owner-based internet survey and the information was recorded without direct or indirect identifiers. The patients/participants provided their written informed consent to participate in this study.

## Author contributions

BA: Writing – original draft, Writing – review & editing. AP: Conceptualization, Data curation, Investigation, Methodology, Writing – review & editing. AS: Data curation, Formal analysis, Investigation, Methodology, Software, Writing – review & editing. NK: Conceptualization, Investigation, Methodology, Project administration, Supervision, Writing – original draft, Writing – review & editing.
